# Configuration Design and Dynamic Characteristics Analysis of Spacecraft Membrane Sunshield

**DOI:** 10.3390/polym14030609

**Published:** 2022-02-04

**Authors:** Tao Peng, Qiuhong Lin, Bingyan Li, Shuwu Dai, Ani Luo, Qiang Cong, Rongqiang Liu

**Affiliations:** 1 Beijing Institute of Spacecraft System Engineering, Beijing 100094, China; tao11102021@163.com (T.P.); libingyan3@hotmail.com (B.L.); daishuwu@nssc.ac.cn (S.D.); qiangcong@sohu.com (Q.C.); 2 College of Mechanical and Electrical Engineering, Harbin Engineering University, Harbin 150001, China; luoani@hrbeu.edu.cn; 3 State Key Laboratory of Robotics and System, Harbin Institute of Technology, Harbin 150001, China; liurq@hit.edu.cn

**Keywords:** membrane sunshield, dynamics, fundamental frequency, modal test

## Abstract

To meet the needs of large space telescopes, such as light weight, high folding ratio, and low manufacturing cost, a flexible deployable regular hexagonal membrane sunshield is proposed in this paper. Firstly, the dynamic equation of the membrane plane is established by the micro-element method. Then, the response surface method is used to obtain the mathematical model of the fundamental frequency of the membrane sunshield. The factors influencing this model, such as the corner pulling force, the effective circle radius, and the edge arch height, are analyzed. By combining the formula of the fundamental frequency of the membrane sunshield and the effective area ratio of the sunshield, the multi-objective optimization function of the fundamental frequency of the membrane sunshield is obtained. A scaled-down experimental prototype of the membrane sunshield is built, and the modal test is performed on the thin membrane plane with a circular fixed boundary in the middle. Comparing the experimental results with the finite element simulation results, the mode shape and the fundamental frequency are highly consistent. This proves that the model can be used to solve the fundamental frequency of the membrane sunshield under the same boundary.

## 1. Introduction

With the development of the aerospace industry, the diameter of space-borne optical systems is being continuously enlarged. To shield them from sunlight, control the temperature, and suppress stray light, higher requirements are put forward on the space-deployable mechanism’s size, weight, and stability. The traditional sunshield structure is challenged to meet the needs of the load on the launch envelope and mass. In addition, traditional designs may cause problems, such as high processing costs for large-diameter thin-walled structures, large deployment flexibility, poor synchronization of parallel driving, and light leakage at moving parts. Due to the outstanding advantages of flexible configuration, ultra-light weight, low production cost, and high folding radio [1], the membrane structure has gradually been applied to the sunshield.

The International X-ray Observatory, which is a tripartite cooperation among the National Aeronautics and Space Administration (NASA), European Space Agency (ESA), and Japan Aerospace Exploration Agency (JAXA), is equipped with a cylindrical membrane sunshield [2]. The sunshield has a diameter of 4 m and an unfolded length of 12.1 m, mainly driven by the middle unfolding structure. Caltech’s submillimeter-wave telescope is equipped with an inflatable, beveled, cylindrical sunshield. The sunshield is driven by 10 inflatable tubes of different lengths to drive six layers of the membrane to expand, providing rigid support for the entire system [3]. To obtain a larger shading angle, the American company QinetiQ North America has developed a chamfered cylindrical sunshield that uses elastic hinges to unfold the membrane [4]. The James Webb Space Telescope (JWST), jointly developed by NASA, the Canadian Space Agency (CSA), and ESA, is equipped with a diamond-shaped membrane sunshield. The deployment is realized by stretching the corners of each layer of the sunshield [5,6,7]. ATLAST-9.2 is a large-aperture space telescope located at LaGrange point 2 (SEL2), equipped with a flat square sunshield composed of four layers of opaque Kapton membrane, blocking light from the sun, moon, and Earth [8]. The JPL and ATK laboratories of the United States have proposed two regular polygon sunshields for cosmic expansion detectors [9]. The smaller one can expand to 5.5 m in diameter, and the larger one has a diameter of 22 m. ESA designed a circular light sunshield for the global astronomical interferometer [10,11], consisting of 12 H-shaped carbon fiber–aluminum racks, each with a length of 3.8 m and a deployment diameter of up to 10.3 m. Kutter et al. [12] designed an inflatable unfolding cone-shaped sunshield consisting of six trapezoidal surfaces and realized a three-layer membrane unfolding through six T-shaped inflatable tubes. The Original Expansion Explorer (PIXIE) is equipped with a four-layer aluminized/Kapton membrane cone-shaped sunshield [13,14], and the outermost dimension can reach 3.5 m after deployment. The large space telescope ST-9 has a beveled conical sunshield [15], which is unfolded with six telescopic tubes of different lengths, and the maximum width after unfolding is greater than 4 m. Tong Zhaoyuan from the Qian Xuesen Space Technology Research Office designed a regular hexagonal membrane sunshield by combining bionic technology and origami technology and verified the feasibility of the structure through finite element simulation [16]. The configuration of sunshields can be divided into flat sunshields and non-planar sunshields. Non-planar sunshields are often used in spacecrafts that change positions with the sun and are not adapted to the sunlight avoidance of large space telescopes. Flat membrane sunshields are mainly used in large space telescopes, without the need for back-and-forth rotation of the sunshield. The non-planar sunshield has a longer history and more applications. However, with the rapid development of the aerospace industry, large flat membrane sunshields will become the primary research trend in the future.

Compared with the configuration design of the membrane sunshield, there are more studies on the membrane structure’s dynamic characteristics and experimental analysis. Shen et al. [17] conducted modal analysis on the membrane with arc edge using simulation software. They obtained the law that the square of the tensile force is proportional to the first-order frequency of the membrane. However, they did not reveal the corresponding relationship between tension and frequency from theoretical analysis. Andreaus et al. [18] investigated the mesh-size independency of second-gradient numerical solutions with respect to the classical first-gradient one. The necessity of second-gradient modeling is finally shown. Zhang Yuelin et al. [19] conducted membrane modal experiments with high-power pulsed lasers as excitation sources. They verified that the flexible piezoelectric element and non-contact vibration test system effectively suppress and evaluate the vibration responses of smart membrane structures. Based on the thin plate theory, Li et al. [20] derived the free vibration equation of the membrane and studied the dynamic response of the rectangular tensioned membrane. Liu et al. [21,22] studied a wave-based active vibration control method for membrane structures and used cable actuators for vibration control. Fang et al. [23,24,25] of NASA measured the vibration characteristics of a 1.5 m × 1.5 m liquid crystal polymer copper-coated thin membrane, which was preloaded by a constant-force spring, excited by a speaker, and measured using a laser non-contact vibration measuring instrument. Flint et al. [26,27] clamped the vertex of the membrane to be tested, mounted it on the backing plate together with the spring-loading device, and transmitted the excitation motion to the membrane by exciting the backing plate. Matsushita et al. [28] used a pulley to guide the load applied at the edge of the membrane in the direction of gravity. The exciter directly touches the middle of the membrane and performs sinusoidal logarithmic scanning excitation on the membrane. During the test, the membrane was placed vertically to avoid the interference of gravity in the out-of-plane direction of the membrane. Iwasa et al. [29] studied the mechanical properties of the flat membrane, wrinkled membrane, and spliced membrane under corner tension by loading the membrane with weights and proposed a method to calculate the upper limit spectrum by measuring several selected points. Meng et al. [30] conducted uniaxial and biaxial stress relaxation tests on the coated fabrics by motor loading and studied the stress relaxation behavior of the membranes over time. Liu et al. [31] used a motor to load the membrane to suppress the vibration of the wrinkled square membrane and excited the frame supporting the membrane by hammering. For the complex membrane structure, its dynamic analysis still relies on finite element simulation for calculation, and there is no complete theoretical formula to solve it. The current dynamic test of membrane structure is performed on a completely flat membrane. However, the sunshield often has a circular void in the middle of the membrane plane. For such flat membranes, there is currently no relevant kinetic experimental study.

Based on the above problems in the configuration design and dynamic analysis of the membrane sunshield, this paper proposes a new type of regular polygon membrane sunshield and conducts a dynamic analysis. Firstly, according to the working principle of the membrane sunshield, the conceptual model of the sunshield is proposed, and the basic configuration of the membrane sunshield is determined. Through the micro-element method, the dynamic equation of the membrane sunshield is established, and the influencing factors that affect the fundamental frequency of the membrane sunshield are analyzed. According to the response surface method, the mathematical model of the fundamental frequency of the membrane sunshield considering the influencing factors is established, and the influence of each variable on the fundamental frequency of the membrane sunshield is analyzed. Combining the fundamental frequency mathematical model and the effective area ratio, the multi-objective optimization function of the fundamental frequency of the membrane sunshield is established. A scale test prototype of the membrane sunshield is built, and modal tests are carried out. The experimental results are compared with the finite element simulation results, and the accuracy of the finite element simulation results under the same boundary is verified. Through the research of this paper, the theoretical formula of the fundamental frequency solution of this kind of membrane sunshield is proposed. It provides theoretical support for the fundamental frequency solution of membrane sunshields in the future.

## 2. Configuration Design of Membrane Sunshield

**Figure 1:**
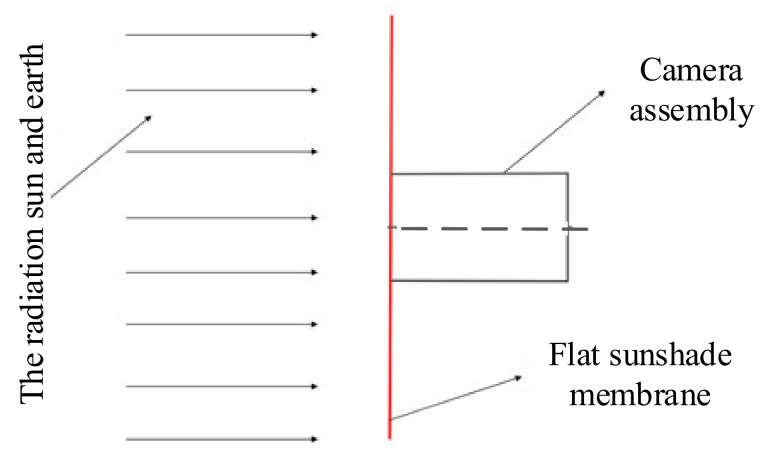
Schematic diagram of the working of the sunshield.

The main function of the sunshield is to eliminate the impact of direct sunlight on the telescope and create a low-temperature background environment. By reducing the camera’s ambient temperature, it provides temperature support for the completion of the camera’s task. The membrane sunshield designed in this paper is located at the LaGrange L2 point. To detect farther and more accurately, a large mirror area is needed to collect more photons, and an open design without a lens barrel is required. The giant membrane blocks the adverse effects of radiation from the sun and Earth, and its light spread is shown in Figure 1. The sunlight and Earth’s radiation sources are located on the same side of the space telescope. The flat sunshade membrane is located between the light source and the space telescope, ensuring that the camera assembly is in an environment free from stray light.

**Figure 2:**
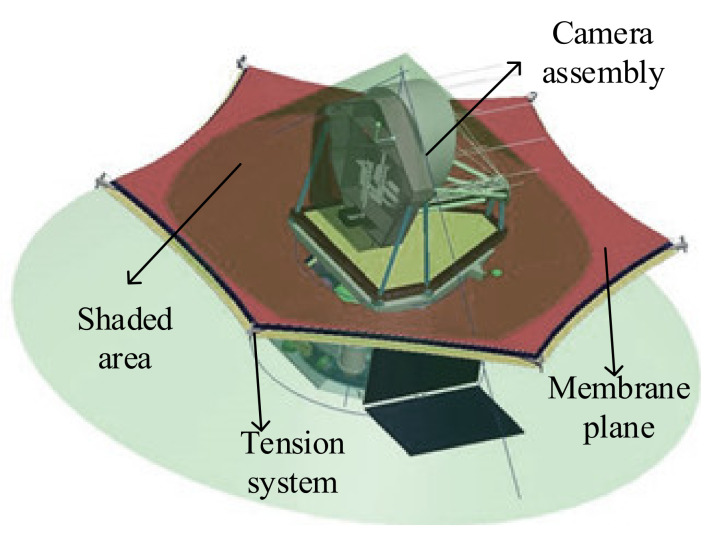
The working principle of the camera.

The size of the sunshield is mainly determined by the position, volume, and field of view of the camera assembly. The observation field of a general telescope is −20°~+20°. According to the current research status of membrane sunshields at home and abroad, this paper designs a regular hexagonal flat membrane sunshield. Its working principle is shown in Figure 2. The middle area of the membrane is fixed on the central hub, and tension is applied to each corner of the membrane to expand the membrane plane. The shaded area in the figure is the effective working surface of the sunshield. The entire camera assembly is in the shadow area determined by the maximum declination angle of sunlight, which effectively avoids the influence of light on the camera assembly.

## 3. Dynamic Analysis of Membrane Sunshield

### 3.1. Free Vibration Equation

**Figure 3:**
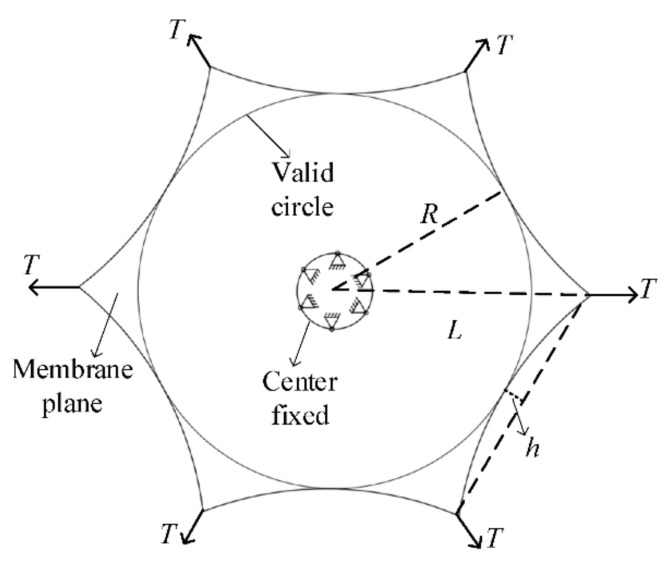
Equivalent mechanical model of membrane sunshield.

In the free vibration analysis of the membrane sunshield, its theoretical mechanical model should be established first. According to the working principle above, the middle of the membrane sunshield is a circular void and is fixed on the central hub. The stiffness of the central hub is much greater than that of the membrane, and the middle of the membrane can be regarded as a fixed constraint without considering the coupled vibration between the two. Ropes tension each corner of the membrane to provide pre-stress in the entire membrane. The membrane arc edge design can effectively improve the mechanical properties of the membrane plane [32] and its equivalent mechanical model is shown in Figure 3. The inscribed circle in the figure is the effective working surface of the sunshield with radius *R*. The distance between the center of the membrane and the corner is *L*, and the arc edge arch height is *h*. The tensile force at each corner point is *T*, and the central circle of the membrane has a fixed constraint.

**Figure 4:**
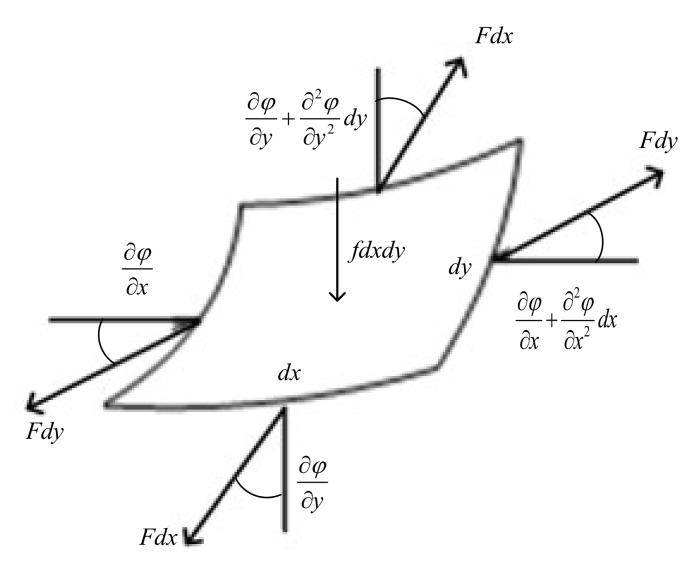
The force analysis diagram of the thin membrane micro-element.

In order to establish the kinetic equation of the membrane sunshield, rectangular micro-elements with lengths *d_x_* and *d_y_* are taken at any point of the membrane. The lateral deflection along the Z-axis of the membrane is *φ*(*x*,*y*,*t*), and *t* is time. The membrane thickness is *b*, the density is *ρ*, and the tension force per unit length is *F*. The distributed force per unit volume along the Z-axis is *f*. The force analysis of its micro-element body is shown in Figure 4.

Then, the dynamic equation of the micro-body along the Z-axis can be expressed as:

(1) $$Fdy\left[\left(\frac{\partial \varphi }{\partial x}+\frac{\partial^{2}\varphi }{\partial x^{2}}dx\right)-\frac{\partial \varphi }{\partial x}\right]+Fdx\left[\left(\frac{\partial \varphi }{\partial y}+\frac{\partial^{2}\varphi }{\partial y^{2}}dy\right)-\frac{\partial \varphi }{\partial y}\right]+fdxdy=\rho bdxdy\frac{\partial^{2}\varphi }{\partial t^{2}},$$

The lateral vibration equation of the membrane is derived as:

(2) $$F\left(\frac{\partial^{2}\varphi }{\partial x^{2}}+\frac{\partial^{2}\varphi }{\partial y^{2}}\right)+f=\rho b\frac{\partial^{2}\varphi }{\partial t^{2}},$$

When considering the free vibration of the membrane, let *f* = 0; then,

(3) $$F\left(\frac{\partial^{2}\varphi }{\partial x^{2}}+\frac{\partial^{2}\varphi }{\partial y^{2}}\right)=\rho b\frac{\partial^{2}\varphi }{\partial t^{2}},$$

The lateral deflection *φ*(*x*,*y*,*t*), of the membrane along the Z-axis can be separated:

(4) $$\varphi \left(x,y,t\right)=\alpha \left(x,y\right)\beta \left(t\right),$$

where *α*(*x*,*y*), is the modal function of the membrane vibration (mm); *β*(*t*) is the generalized coordinate of the membrane vibration. Substituting Equation (4) into Equation (3):

(5) $$\frac{F}{\alpha }\left(\frac{\partial^{2}\alpha }{\partial x^{2}}+\frac{\partial^{2}\alpha }{\partial y^{2}}\right)=\frac{\rho b}{\beta }\frac{\partial^{2}\beta }{\partial t^{2}},$$

The left side of Equation (5) is independent of *t*, and the right end of the above equation is independent of *x*,*y*. The left and right ends of the equation are always equal to a constant, denoted as $-\omega^{2}$, and two linear ordinary differential equations are obtained:

(6) $$\frac{\partial^{2}\alpha }{\partial x^{2}}+\frac{\partial^{2}\alpha }{\partial y^{2}}+\frac{\rho b\omega^{2}\alpha }{F}=0,$$

(7) $$\frac{\partial^{2}\beta }{\partial t^{2}}+\omega^{2}\beta =0,$$

The solution of Equation (7) is a single-degree-of-freedom linear vibration equation, and its general solution is:

(8) $$\beta \left(t\right)=A\sin\left(\omega t+\theta \right),$$

The solution of Equation (6) mainly depends on the shape of the membrane, the boundary conditions, and the properties of the membrane itself.

Combined with Equation (6) and the shape of the membrane sunshield, the fundamental frequency of the membrane sunshield mainly depends on the membrane tension *F*, density *ρ*, thickness *b*, effective circle radius *R*, arc edge arch height *h*, and central hub size. The sunshield is usually composed of a 0.5-mm-thick polyimide membrane with a density of 1420 kg/m^3^. The central hub of the sunshield is determined by the camera load, and the positioning radius here *r* is 0.5 m. The tension *F* of the membrane is provided by the corner tension *T*. The influencing factors of the membrane sunshield are mainly determined by three variables: the tensile force *T*, the effective circle radius *R*, and the arc edge arch height *h*.

### 3.2. Fundamental Frequency Mathematical Model Establishment

Due to the complexity of the membrane boundary of the membrane sunshield, it is not easy to directly apply the theoretical derivation method to generate a reasonable mathematical model. Each product design relies heavily on the calculation of simulation software, the process is relatively cumbersome, and the product development cycle is prolonged. It is preferable to use the response surface method to establish a sunshield fundamental frequency mathematical model.

The key to the success of the response surface lies in the establishment of the experimental method, and the most widely used method is the full factor method. In this section, an orthogonal test is designed for the membrane sunshield by the full factor method. The fundamental frequency of the sunshield corresponding to different parameters is obtained through finite element simulation. The method of multivariate nonlinear regression is used to obtain reasonable coefficients. The variation range of the three design parameters in this paper is as follows: the effective circle radius from 4 m to 7.5 m, the arch height from 0 m to 1.2 m, and the tensile force from 0 N to 80 N. Each factor takes eight tries, and, finally, 512 results are obtained. The analysis results are shown in Appendix A.

At present, the selection of empirical formulas generally adopts the following polynomials:

$$1,\;x_{1},\;x_{2},\;x_{3},\;\ldots ,\;x_{n}\;x_{1}^{2},\;x_{1}x_{1},\;x_{1}x_{2},\;x_{1}x_{3},\;\ldots ,\;x_{1}x_{n},\;\ldots ,\;x_{n}^{2}\;x_{1}^{3},\;x_{1}^{2}x_{2},\;\ldots ,\;x_{1}^{2}x_{n},\;x_{1}x_{2}^{2},\;x_{1}x_{3}^{2},\;\ldots ,\;x_{1}x_{n}^{2},\;\ldots ,\;x_{n}^{3}$$

**Figure 5:**
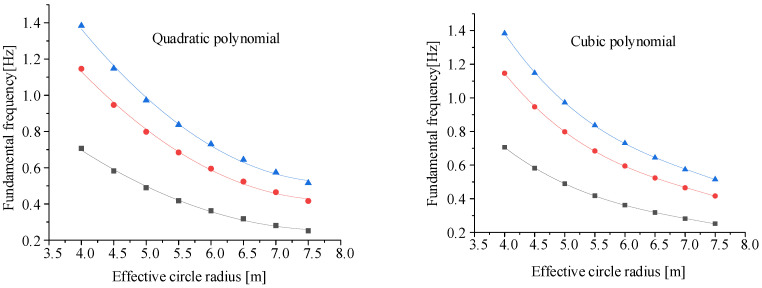
Fitting of effective circle radius to fundamental frequency.

**Figure 6:**
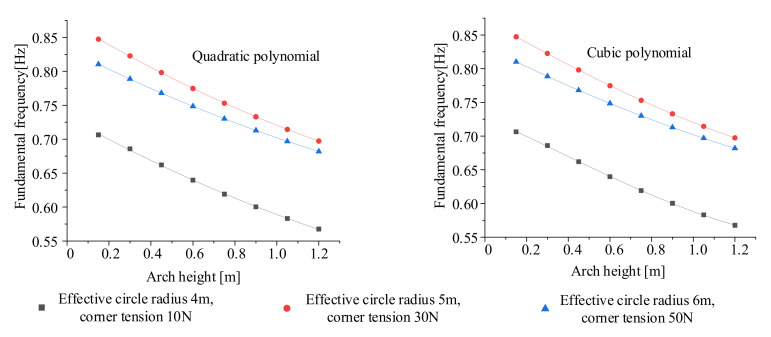
Fitting of arch height to fundamental frequency.

**Figure 7:**
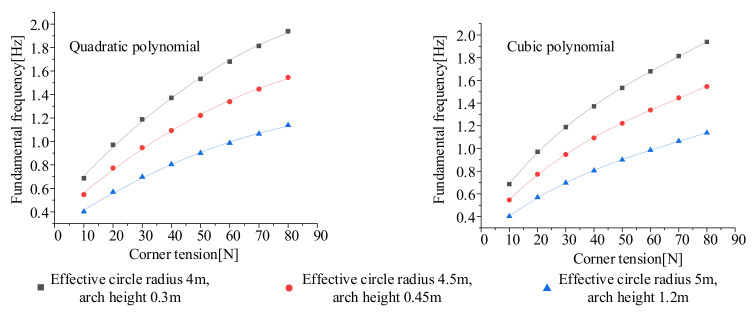
Fitting of corner tension to fundamental frequency.

There are three variables in this experiment. In theory, the least-squares method can be used to fit the data of the quadratic polynomial. However, the data calculated by the fitting formula are quite different from the original data, so it cannot be used as an empirical formula. A reasonable polynomial needs to be selected for fitting. To ensure the accuracy of the final fitting formula, the control variable method is adopted to explore the influence of the effective shading radius *R*, the arch height *h*, and the corner tension *T* on the fundamental frequency of the sunshield. To avoid the possibility of data affecting the results, each impact factor selects three sets of data for fitting, and the fitting results are shown in Figure 5, Figure 6 and Figure 7.

According to the fitting of the above three variables to the fundamental frequency, the cubic polynomial fitting is more accurate when the effective circle radius and the force are fitted to the fundamental frequency. When the arc edge arch height is fitted to the fundamental frequency, the quadratic polynomial and the cubic polynomial have the same accuracy. The quadratic term can be taken as the fitting polynomial to save computing resources.

Then, the variables *R*, *h*, and *T* of the membrane sunshield affect the fundamental frequency as follows:

(9) $$\begin{array}{l} q=\propto \left(k_{1}R^{3}+k_{2}R^{2}+k_{3}R+k_{4}\right)\\ q=\propto \left(k_{5}h^{2}+k_{6}h+k_{7}\right)\\ q=\propto \left(k_{8}T^{3}+k_{9}T^{2}+k_{10}T+k_{11}\right) \end{array},$$

There is no coupling relationship between the effective circle radius *R*, the arch height *h*, and the corner tension *T* independently. Therefore, it can be concluded that the mathematical model of the fundamental frequency of the membrane sunshield is:

(10) $$q=\left(k_{1}R^{3}+k_{2}R^{2}+k_{3}R+k_{4}\right)\left(k_{5}h^{2}+k_{6}h+k_{7}\right)\left(k_{8}T^{3}+k_{9}T^{2}+k_{10}T+k_{11}\right)+k_{12}$$

The data in Appendix A are fitted by the method of multiple linear regression. The coefficients can be obtained as shown in Table 1.

Then, the fundamental frequency can be expressed as:

(11) $$\begin{array}{l} q=\left(-8.1\times 10^{-3}R^{3}+1.77\times 10^{-1}R^{2}-1.37R+4.09\right)\left(5.87\times 10^{-6}h^{2}-3.62\times 10^{-5}h+1.7\times 10^{-4}\right)\\ \left(1.09\times 10^{-2}T^{3}-2.28T^{2}+2.49\times 10^{2}T+2.32\times 10^{3}\right)+1.55\times 10^{-2} \end{array}\mathrm{Hz}$$

(12) $$t=\frac{q-q_{0}}{q}\times 100,$$

The fitted formula needs to have sufficient precision to be used as an empirical formula for future product design. Equation (12) can be used for error analysis to verify the formula’s accuracy. Five groups of data are randomly selected within the variable range for simple experimental verification, and the results are shown in Table 2.

It can be seen from the above five sets of results that the errors of the mathematical model fitted above are relatively small within the value range of the three variables. This shows that it is accurate within the value range of the three variables, which can provide a reference for future product design.

### 3.3. The Law of Variable Influence on Fundamental Frequency and Multi-Objective Optimization

**Figure 8:**
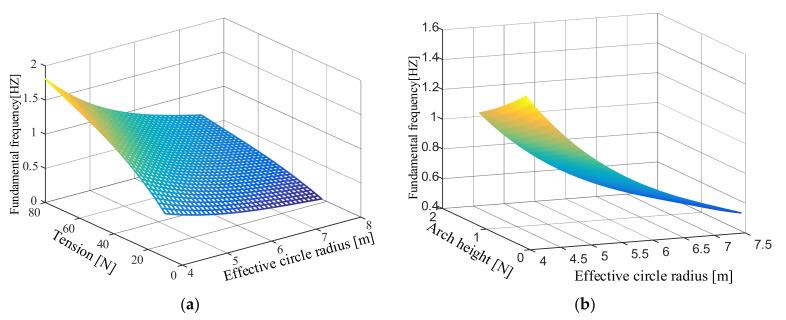

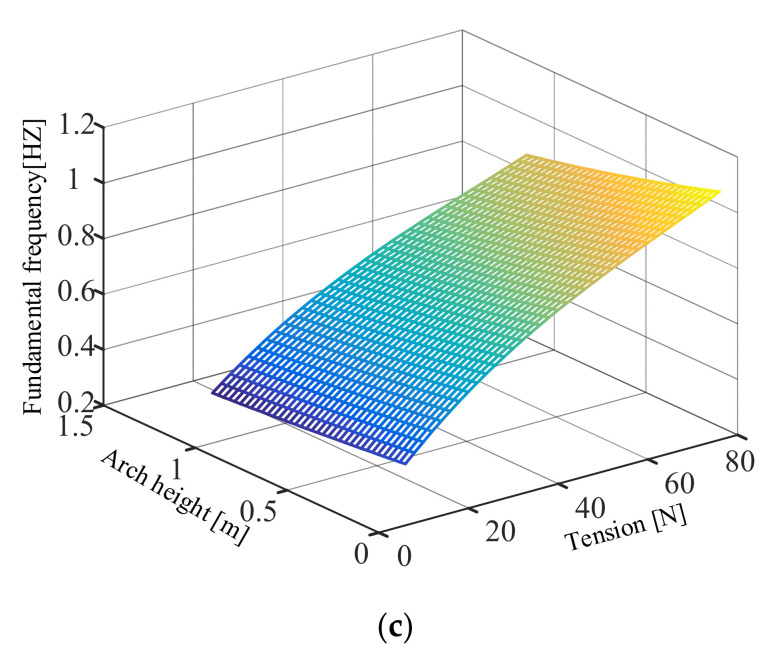
The law of the influence of two variables on the fundamental frequency. (**a**) The arch height is 0.6 m; (**b**) the tension is 30 N; (**c**) the effective circle radius is 5.5 m.

The observation of the data shows that the increase in the arch height and the effective circle radius causes the fundamental frequency to decrease. The increase in the pulling force causes the fundamental frequency to rise. However, the specific function changes of these factors are not yet clear. Since the three variables jointly affect the fundamental frequency of the membrane, one of the variables should be kept as a fixed value in the process of analyzing its variation law. According to the fundamental frequency formula fitted above, the law of the influence of two variables on the fundamental frequency is analyzed. Its three-dimensional map is shown in Figure 8.

It can be seen from the figures that when the arch height is fixed, the fundamental frequency of the membrane plane increases as the effective circle radius decreases or the tension increases. When the tension is fixed, the fundamental frequency of the membrane plane increases as the arch height decreases and the effective circle radius decreases. Therefore, a higher fundamental frequency can be obtained by increasing the corner point pulling force, reducing the arch height of the arc edge, and reducing the effective circle radius.

The membrane arc edge design can improve the membrane’s stress distribution state. An increase in the effective circle radius provides a larger shading plane. However, both will have a certain impact on the fundamental frequency and effective area ratio of the membrane sunshield. In order to resolve this contradiction, it is necessary to establish a multi-objective optimization function of the fundamental frequency of the membrane sunshield.

**Figure 9:**
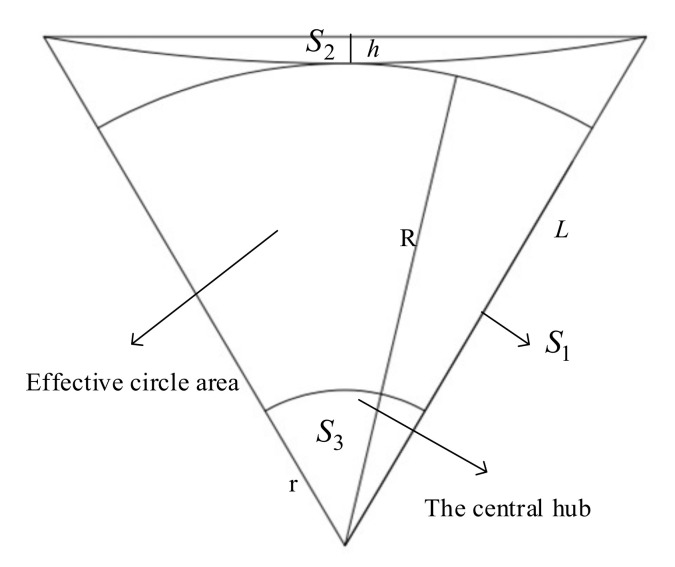
Simplified model of membrane configuration.

The area of the sunshield is mainly divided into three parts: the area $S_{1}$ of the regular hexagon, the area $S_{2}$ beyond the arc side, and the area $S_{3}$ of the central hub. A simplified model of its configuration is shown in Figure 9.

According to the geometric relationship, the radius $R_{1}$ of the arc edge is:

(13) $$R_{1}=\frac{h^{2}+{\left(L/2\right)}^{2}}{2h},$$

The central angle *γ* of the circle corresponding to the arc edge is obtained as:

(14) γ=arctanL2R1−h,

The area $S_{2}$ outside the arc edge can be obtained as:

(15) $$S_{2}=6\gamma R_{1}^{2}-3L\cdot \frac{{\left(L/2\right)}^{2}-h^{2}}{2h},$$

The area $S_{1}$ of a regular hexagon can be obtained as:

(16) $$S_{1}=6\times \frac{1}{2}\cdot \sin60\cdot L^{2}=\frac{3\sqrt{3}}{2}L^{2},$$

The area $S_{3}$ of the center hub can be obtained as:

(17) $$S_{3}={\pi r}^{2},$$

The area *S* of the membrane sunshield is:

(18) $$S=S_{1}-S_{2}-S_{3},$$

The effective area $S_{0}$ of the membrane sunshield is:

(19) $$S_{0}=\pi R^{2}-{\pi r}^{2},$$

The distance *L* from the center of the membrane to the corner point is:

(20) $$L=\frac{2\sqrt{3}}{3}\left(R+h\right),$$

The effective area ratio of the membrane plane is:

(21) $$p=\frac{S_{0}}{S},$$

Combined with the fundamental frequency formula, the effective area ratio, and the value range of each variable, the optimization function is determined as follows.

(22) $$\left\{\begin{array}{l} q=\left(-8.1\times 10^{-3}R^{3}+1.77\times 10^{-1}R^{2}-1.37R+4.09\right)\\ \left(5.87\times 10^{-6}h^{2}-3.62\times 10^{-5}h+1.7\times 10^{-4}\right)\\ \left(1.09\times 10^{-2}T^{3}-2.28T^{2}+249.26T+2317.74\right)+1.55\times 10^{-2}\\ p=\frac{S_{0}}{S}\\ 4\;m\leq R\leq 7.5\;m\\ 0\ll h\leq 1.2\;m\\ 5\;N\leq T\leq 80\;N\\ c_{1}\leq q\leq c_{2},\;c_{3}\leq p \end{array}\right.,$$

where $c_{1}$ and $c_{2}$ are the expected fundamental frequency ranges for the thin membrane sunshield design $\;c_{3}$. is the minimum effective area ratio designed for the membrane sunshield.

## 4. Dynamic Test Analysis of Membrane Sunshield

### 4.1. Sample Preparation

**Figure 10:**
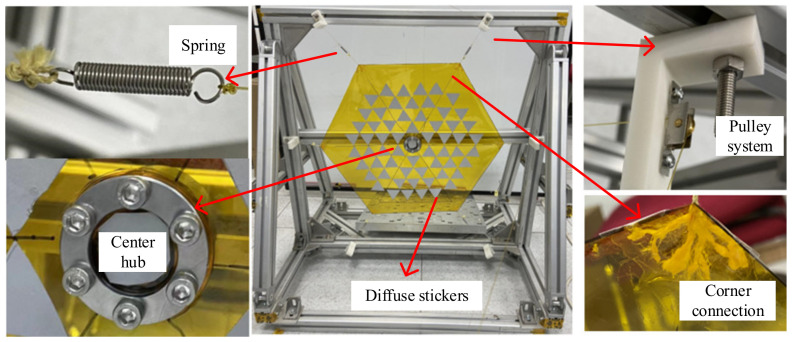
Assembly of the test piece of the membrane sunshield.

Modal tests have strict demands on the boundaries and surrounding environment of the membrane. To ensure the test’s accuracy, the membrane’s test environment should be kept consistent with the simulation environment as much as possible. According to the working principle, the membrane is in a fixed state relative to the central cylinder, and the six corners of the membrane are opened under the tension. Based on the above membrane boundaries, a modal test platform for the membrane sunshield is built in this paper, as shown in Figure 10.

The test platform consists of an external support frame, pulley system, spring, center cylinder, and diffuse reflection paper. The support frame in the figure provides a support platform for the entire sample. The fundamental frequency of the frame should be larger than that of the membrane to avoid coupling between the fundamental frequency of frame and membrane. The pulley system mainly changes the direction of the pulling force and reduces the influence of the friction force on the fundamental frequency test. Both ends of the rope are attached to the spring and weight, respectively, to provide tension to the membrane. The rope is divided into several thin ropes, which are then secured over the corners of the membrane with scotch tape and partially secured at the ends of the ropes. The spring provides constant tension for the membrane to ensure that each corner receives the same tension. The central cylinder is divided into upper and lower parts, and the membrane is sandwiched and fixed by a flange connection. The diffuse paper reflects the laser light, allowing the vibrometer to collect the returned light.

In this paper, the scaled prototype of the membrane sunshield is selected for the modal test. The membrane size is a regular hexagonal membrane with a side length of 260 mm. There is a circular void with a diameter of 50 mm in the middle of the membrane. The membrane thickness is 0.05 mm, the material is polyimide, and the density is 1420 kg/m^3^. The envelope dimensions of the frame are 760 mm × 760 mm × 640 mm. The entire frame is fixed to the ground with weights to increase the fundamental frequency of the frame.

### 4.2. Experiment Design

According to the shape of the membrane, the plane test points of the membrane are laid out. Each sticker is an equilateral triangle with a side length of 30 mm. There are 60 stickers on the entire membrane surface, which ensures the accuracy of the membrane fundamental frequency test. Since the stress distribution at the edge of the membrane is relatively inhomogeneous, which will increase the instability of the membrane’s mode shape, no measurement points are attached. In the experiment, the membrane is excited by the external beating frame so that the membrane can vibrate freely. Each measurement point is sampled for 8 s, the frame is tapped every 4 s, and a single measurement takes 8 min. We provide 2 N and 4 N tensile force to the membrane, respectively. The vibration frequency curves of the membrane under two tension forces are measured.

**Figure 11:**
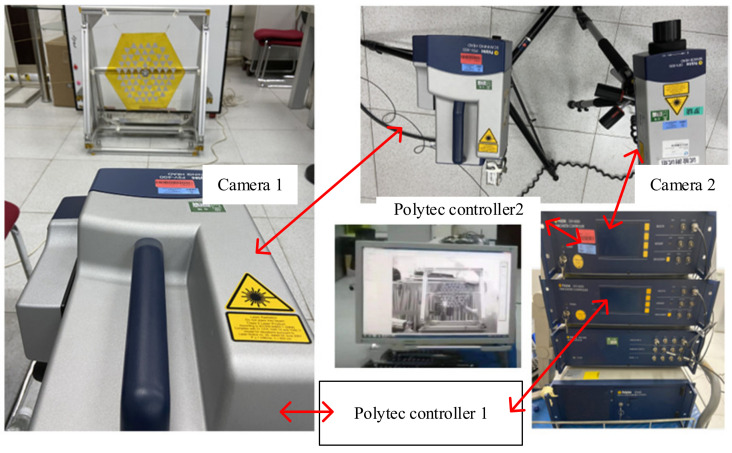
Schematic diagram of the frequency test of membrane.

The instrument used to test the vibration of the membrane in this paper is the Polytec laser vibrometer. The membrane vibration test equipment is shown in Figure 11. The system contains a total of two cameras. Camera 1 is used as the main camera to collect vibration speed information. Camera 2 serves as an auxiliary camera to provide the position reference of the system. The Polytec controller 1 controls laser camera 1 to scan the target. The Polytec controller 2 controls camera 2 to pick up a sticker in the membrane to provide a position reference.

### 4.3. Test Analysis

#### 4.3.1. Finite Element Simulation Analysis

When the membrane is modally tested in air, the effect of the air’s additional mass on the membrane’s frequency should not be ignored. The article [33] gives the free vibration equation of the membrane in air.

(23) $$-\omega^{2}\left(1+\alpha_{a}\right)M\Phi +K\Phi =0,$$

where $\alpha_{a}$ is the added-mass ratio denoting the ratio of added air mass to the mass of the membrane vibrating in air. M represents the mass matrix, K is the stiffness matrix, and Φ is the mode shape function.

Minami [34] applied the thin-wing theory to study the membrane vibration in the air and pointed out that the added-mass ratio $\alpha_{a}$ does not depend on the period and amplitude of the oscillation. One of his conclusions is that the height of an equivalent air layer corresponding to the added mass distributed uniformly over the membrane is equal to 68% of the membrane length, which is confirmed by comparison with the results obtained from a source-distribution Green’s function approach. The formula for the additional mass ratio given by Minami is:

(24) $$\alpha_{a}=0.68\rho_{a}l_{m}/m_{m},$$

In the formula, $\rho_{a}$ represents the air density (kg/m^3^); $l_{m}$ is the membrane length, and it refers to the distance between the fixed boundaries of the membrane (m); $m_{m}$ is the mass of the membrane (kg).

Taking the air density in the atmosphere as 1.18 kg/m^3^, the added-mass ratio can be obtained as 13 according to the size of the membrane mentioned in Section 4.1. Therefore, the density of the membrane should be set to 1420 kg/m^3^ × (1 + 13) in the finite element simulation. The analytical element is a three-node triangular membrane element in Abaqus (M3D3), with 2 N and 4 N tensile forces applied to the membrane corners, respectively. The central circle of the membrane is a fixed constraint, and the analysis results are shown in Table 3.

#### 4.3.2. Prototype Test Analysis

**Figure 12:**
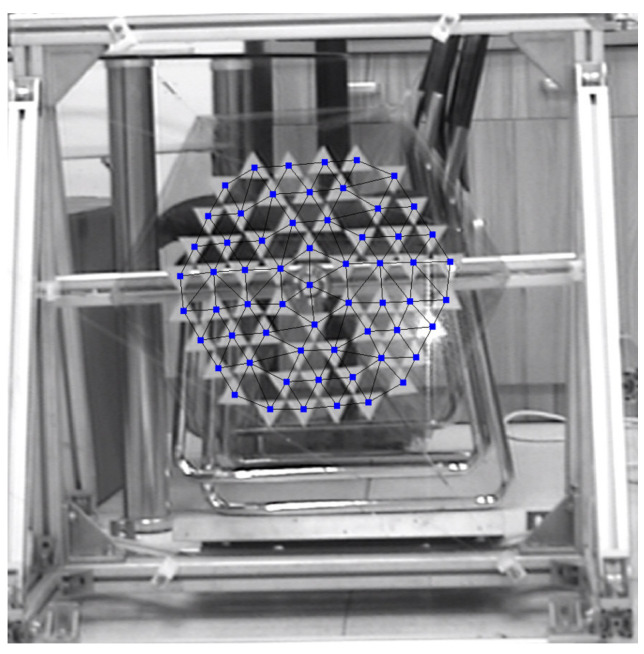
Meshing of thin membrane test points.

A total of 60 measuring points were added to the scanning points in the computer. The camera laser accurately illuminates each sticker, and the 60 measuring points are connected to divide the grid, as shown in Figure 12.

**Figure 13:**
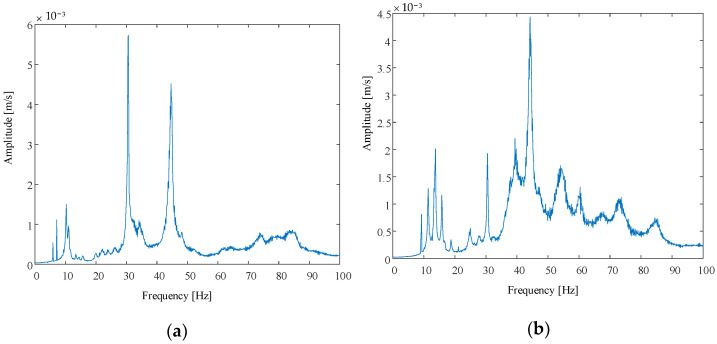
Amplitude–frequency characteristic curve when the tensile force is 2 N and 4 N, respectively. (**a**) The tensile force is 2 N; (**b**) the tensile force is 4 N.

The tests were carried out under two working conditions, 2 N and 4 N, respectively. The amplitude–frequency characteristic curve obtained is shown in Figure 13.

According to the analysis results of the above figure and the finite element simulation analysis, the results are shown in Table 3.

From the comparison of the above two results, the overall frequency of the membrane tends to increase with the increase in tensile force. When the tensile force is 2 N, the errors of the first-order frequency and the second-order frequency of the membrane are both less than 12%, which are all within the acceptable range. When the pulling force is 4 N, the second-order frequency error reaches 21%. The increase in error results from the coupling between the fundamental frequency of the support frame and the frequency of the membrane, as the latter increases when the pulling force is larger.

**Figure 14:**
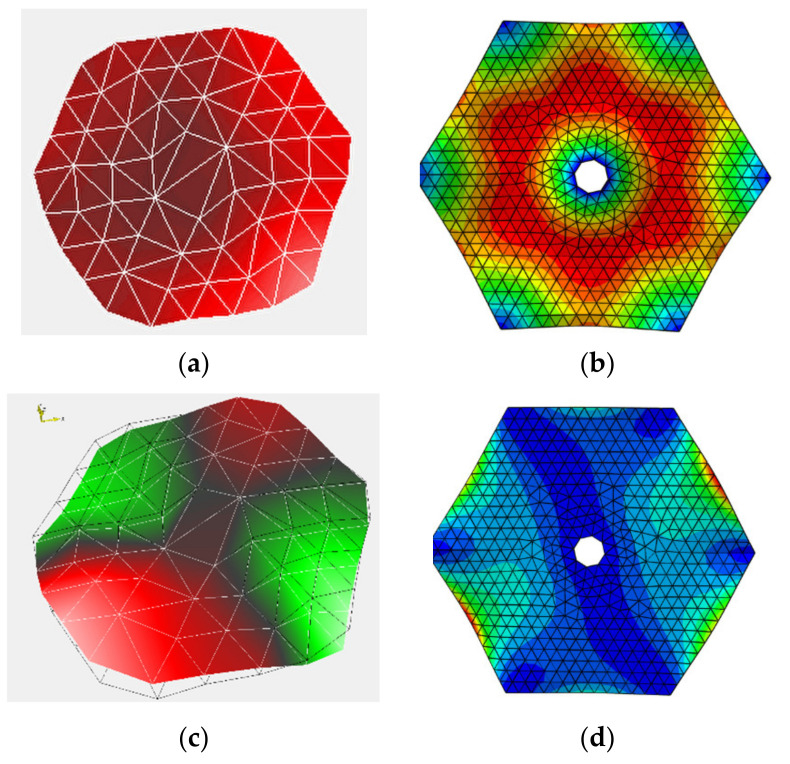
Comparison of test mode shape and simulation mode shape. (**a**) Test first mode shape; (**b**) simulated first-order mode shapes; (**c**) test second mode shape; (**d**) simulated second-order mode shapes.

Since the boundary conditions and membrane dimensions under 2 N tension and 4 N do not change, the mode shapes of the two modes are the same. The mode shapes of the test results and the simulation results are shown in Figure 14.

The Polytec Vibrometer uses a linear interpolation calculation method to process the data, and it automatically fills in the circular voids in the middle of the membrane. Therefore, there are no circular vacancies in the middle of the membrane in Figure 14a,c. The edge of the mode shape obtained by the finite element simulation is slightly different from that in the test state, mainly because the edge mode shape of the membrane is not measured in the test state. The two modes of vibration maintain a similar configuration. Membranes have lower amplitudes in the middle of the first mode and relatively higher amplitudes at the periphery. The intermediate amplitude of the thin membrane in the experimental state is higher than that in the finite element simulation, which is mainly due to the interpolation calculation used by the experimental software. The second-order model is a torsional mode, similar to a “V” shape. This shows that under the same boundary conditions, the fundamental frequency of the membrane sunshield can be solved by finite element simulation.

## 5. Conclusions

According to the working principle of the space telescope at the L2 point, this paper proposes a flexible and deployable regular hexagonal membrane sunshield. According to the working form of the membrane sunshield, the equivalent mechanical model of the membrane sunshield is given. Then, the dynamic equation of the membrane is deduced by the method of rectangular micro-elements. The analysis shows that the three variables of corner tension, effective circle radius, and arc edge arch height are the main factors affecting the fundamental frequency of the membrane sunshield. Using the response surface method, the mathematical formula of the fundamental frequency of the membrane sunshield is obtained, with the effective circle radius from 4 m to 7.5 m, the arch height from 0 m to 1.2 m, and the tensile force from 0 N to 80 N. Five sets are randomly selected to verify the fundamental frequency formula, and the error range is within 2.5%, which proves the formula’s accuracy. The variation law of the fundamental frequency of the membrane under the influence of two variables is analyzed. The multi-objective optimization function of the membrane sunshield is established based on the parameter analysis in terms of the arc edge arch height, the effective circle radius, the fundamental frequency, and the effective area ratio. A test prototype of the membrane sunshield is built, and the modal test of the membrane sunshield in the atmospheric environment is carried out. By comparing the experimental results with the finite element simulation results, it is found that the fundamental frequency and mode shape of the two are highly consistent. The test shows that under the same boundary, the fundamental frequency of the membrane sunshield can be solved by finite element simulation. It is proven that the fundamental frequency mathematical model can be used to solve the fundamental frequency of the membrane sunshield.

## Figures and Tables

**Table 1:** The coefficients of the base frequency formula of the sunshield.

$k_{1}$	$k_{2}$	$k_{3}$	$k_{4}$	$k_{5}$	$k_{6}$
−8.1 × 10^−3^	1.77 × 10^−1^	−1.37	4.09	5.87 × 10^−6^	3.62 × 10^−5^
$k_{7}$	$k_{8}$	$k_{9}$	$k_{10}$	$k_{11}$	$k_{12}$
1.7 × 10^−4^	1.09 × 10^−2^	−2.28	2.49 × 10^2^	2.32 × 10^3^	1.55 × 10^−2^

**Table 2:** Comparison of theoretical and simulated values of fundamental frequency.

Effective Circle Radius (m)	Arch Height (m)	Corner Tension (N)	Theoretical Value $\times 10^{-1}$ (Hz)	Simulation Value $\times 10^{-1}$ (Hz)	Error
4.2	0.2	15	7.76	7.90	1.8%
5.2	0.4	25	6.87	6.90	0.44%
5.8	0.6	35	6.59	6.61	0.30%
6.4	0.8	45	6.18	6.21	0.49%
7.2	1	55	5.45	5.57	2.2%

**Table 3:** Comparison of simulation analysis results and test results.

Working Condition	Frequency Order	Simulation Frequency (Hz)	Test Frequency (Hz)	Error
2 N	First-order frequency	5.76	6.25	7.8%
	Second-order frequency	6.35	7.15	11.2%
4 N	First-order frequency	8.14	9.45	13.86%
	Second-order frequency	8.98	11.37	21%

## Appendix A

**Table A1:** Finite element simulation experimental data of membrane sunshield.

Effective Circle Radius	Arch Height	Fundamental Frequency under Different Tension Levels (Hz)							
		10/N	20/N	30/N	40/N	50/N	60/N	70 N	80 N
4000	150	0.70635	0.99888	1.2233	1.4125	1.5792	1.7298	1.8683	1.9972
	300	0.68585	0.96989	1.1878	1.3715	1.5333	1.6796	1.8140	1.9392
	450	0.66203	0.93620	1.1465	1.3238	1.4800	1.6212	1.7510	1.8718
	600	0.63965	0.90455	1.1078	1.2791	1.4300	1.5664	1.6918	1.8085
	750	0.61907	0.87544	1.0721	1.2379	1.3839	1.5159	1.6373	1.7502
	900	0.60028	0.84887	1.0396	1.2003	1.3419	1.4699	1.5875	1.6970
	1050	0.58320	0.82471	1.0100	1.1661	1.3037	1.4280	1.5423	1.6486
	1200	0.56764	0.80270	0.98301	1.1350	1.2688	1.3898	1.5011	1.6045
4500	150	0.58186	0.82284	1.0077	1.1636	1.3008	1.4249	1.5390	1.6452
	300	0.56496	0.79893	0.97844	1.1298	1.2630	1.3835	1.4943	1.5974
	450	0.54658	0.77294	0.94661	1.0930	1.2219	1.3385	1.4457	1.5454
	600	0.52931	0.74851	0.91669	1.0584	1.1833	1.2962	1.4000	1.4966
	750	0.51345	0.72609	0.88922	1.0267	1.1478	1.2573	1.3580	1.4517
	900	0.49887	0.70546	0.86395	0.99755	1.1152	1.2216	1.3194	1.4140
	1050	0.48548	0.68653	0.84077	0.97077	1.0853	1.1888	1.2839	1.3725
	1200	0.47319	0.66914	0.81947	0.94618	1.0578	1.1587	1.2514	1.3377
5000	150	0.48922	0.69183	0.84728	0.97831	1.0937	1.1981	1.2940	1.3833
	300	0.47495	0.67165	0.82256	0.94976	1.0618	1.1631	1.2562	1.3429
	450	0.46085	0.65170	0.79813	0.92156	1.0303	1.1286	1.2189	1.3031
	600	0.44729	0.63254	0.77466	0.89446	0.99998	1.0954	1.1831	1.2647
	750	0.43481	0.61489	0.75304	0.86949	0.97207	1.0648	1.1501	1.2294
	900	0.42321	0.59848	0.73294	0.84628	0.94612	1.0364	1.1193	1.1966
	1050	0.41251	0.58335	0.71441	0.82488	0.92219	1.0101	1.0910	1.1663
	1200	0.40260	0.56933	0.69724	0.80505	0.90002	0.98586	1.0648	1.1382
5500	150	0.41812	0.59128	0.72414	0.83613	0.93478	1.0240	1.1060	1.1823
	300	0.40647	0.57482	0.70397	0.81284	0.90874	0.99543	1.0751	1.1493
	450	0.39506	0.55868	0.68420	0.79002	0.88322	0.96748	1.0449	1.1170
	600	0.38434	0.54352	0.66564	0.76858	0.85926	0.94123	1.0166	1.0876
	750	0.37430	0.52931	0.64825	0.74850	0.83681	0.91663	0.99003	1.0583
	900	0.36494	0.51608	0.63204	0.72978	0.81588	0.89371	0.96527	1.0319
	1050	0.35624	0.50378	0.61697	0.71238	0.79642	0.87239	0.94224	1.0072
	1200	0.34814	0.49232	0.60293	0.69617	0.77830	0.85253	0.92079	0.98431
6000	150	0.36241	0.51250	0.62765	0.72472	0.81023	0.88752	0.95860	1.0247
	300	0.35278	0.49888	0.61097	0.70546	0.78869	0.86393	0.93311	0.99750
	450	0.34351	0.48577	0.59492	0.68692	0.76797	0.84124	0.90860	0.97130
	600	0.33477	0.47342	0.57979	0.66946	0.74845	0.81985	0.88550	0.94660
	750	0.32657	0.46183	0.56560	0.65307	0.73012	0.79977	0.86382	0.92342
	900	0.31890	0.45097	0.55230	0.63771	0.71295	0.78096	0.84350	0.90170
	1050	0.31173	0.44084	0.53989	0.62338	0.69692	0.76341	0.82454	0.88143
	1200	0.30502	0.43134	0.52826	0.60995	0.68191	0.74696	0.80677	0.86243
6500	150	0.31780	0.44941	0.55040	0.63552	0.71050	0.77829	0.84061	0.89862
	300	0.30979	0.43809	0.53653	0.61950	0.69260	0.75867	0.81943	0.87597
	450	0.30217	0.42732	0.52334	0.60427	0.67557	0.74003	0.79929	0.85445
	600	0.29493	0.41707	0.51079	0.58979	0.65938	0.72228	0.78012	0.83396
	750	0.28815	0.40748	0.49905	0.57623	0.64422	0.70568	0.76219	0.81478
	900	0.28177	0.39847	0.48800	0.56347	0.62996	0.69006	0.74532	0.79674
	1050	0.27578	0.39000	0.47763	0.55150	0.61656	0.67538	0.72947	0.77980
	1200	0.27015	0.38203	0.46787	0.54023	0.60397	0.66159	0.71456	0.76386
7000	150	0.28145	0.39801	0.48744	0.56283	0.62924	0.68927	0.74447	0.79584
	300	0.27477	0.38857	0.47588	0.54948	0.61432	0.67293	0.72682	0.77697
	450	0.26839	0.37955	0.46484	0.53673	0.60007	0.65732	0.70996	0.75895
	600	0.26236	0.37103	0.45440	0.52467	0.58658	0.64255	0.69400	0.74190
	750	0.25666	0.36296	0.44452	0.51327	0.57383	0.62858	0.67892	0.72577
	900	0.25130	0.35538	0.43523	0.50254	0.56184	0.61544	0.66472	0.71059
	1050	0.24624	0.34822	0.42647	0.49242	0.55053	0.60305	0.65134	0.69628
	1200	0.24107	0.34091	0.41751	0.48207	0.53895	0.59037	0.63765	0.68165
7500	150	0.25144	0.35558	0.43548	0.50283	0.56216	0.61580	0.66511	0.71101
	300	0.24580	0.34760	0.42570	0.49154	0.54954	0.60197	0.65018	0.69504
	450	0.24041	0.33998	0.41637	0.48076	0.53749	0.58877	0.63592	0.67981
	600	0.23531	0.33277	0.40754	0.47057	0.52610	0.57629	0.62245	0.66540
	750	0.23049	0.32595	0.39919	0.46093	0.51532	0.56449	0.60970	0.65177
	900	0.22593	0.31950	0.39129	0.45180	0.50511	0.55331	0.59762	0.63886
	1050	0.22161	0.31339	0.38381	0.44317	0.49546	0.54273	0.58620	0.62665
	1200	0.21757	0.30761	0.37673	0.43500	0.48632	0.53272	0.57538	0.61509
